# A Cytosine Methytransferase Modulates the Cell Envelope Stress Response in the Cholera Pathogen

**DOI:** 10.1371/journal.pgen.1005666

**Published:** 2015-11-20

**Authors:** Michael C. Chao, Shijia Zhu, Satoshi Kimura, Brigid M. Davis, Eric E. Schadt, Gang Fang, Matthew K. Waldor

**Affiliations:** 1 Division of Infectious Disease, Brigham and Women’s Hospital, Boston, Massachusetts, United States of America; 2 Howard Hughes Medical Institute, Boston, Massachusetts, United States of America; 3 Department of Microbiology and Immunobiology, Harvard Medical School, Boston, Massachusetts, United States of America; 4 Department of Genetics and Genomic Sciences, Institute for Genomics and Multi-scale Biology, Mount Sinai School of Medicine, New York, New York, United States of America; University of Geneva Medical School, SWITZERLAND

## Abstract

DNA methylation is a key epigenetic regulator in all domains of life, yet the effects of most bacterial DNA methyltransferases on cellular processes are largely undefined. Here, we used diverse techniques, including bisulfite sequencing, transcriptomics, and transposon insertion site sequencing to extensively characterize a 5-methylcytosine (5mC) methyltransferase, VchM, in the cholera pathogen, *Vibrio cholerae*. We have comprehensively defined VchM’s DNA targets, its genetic interactions and the gene networks that it regulates. Although VchM is a relatively new component of the *V*. *cholerae* genome, it is required for optimal *V*. *cholerae* growth *in vitro* and during infection. Unexpectedly, the usually essential σ^E^ cell envelope stress pathway is dispensable in *∆vchM V*. *cholerae*, likely due to its lower activation in this mutant and the capacity for VchM methylation to limit expression of some cell envelope modifying genes. Our work illuminates how an acquired DNA methyltransferase can become integrated within complex cell circuits to control critical housekeeping processes.

## Author Summary

Methylation of DNA is used by numerous organisms to regulate a wide variety of cellular processes, but specific roles for most DNA methyltransferases have not been defined. We studied one such enzyme in *Vibrio cholerae*, the cholera pathogen, using genome-wide approaches to compare DNA methylation, gene expression, and the sets of genes required or dispensable for growth in bacterial strains that produced or lacked this enzyme. These studies allowed us to identify numerous cellular processes regulated, either directly or indirectly, by this cytosine methyltransferase. In particular, we found that an absence of enzyme activity was associated with reduced levels of a bacterial stress response; consequently, a stress response pathway that is essential in wild type bacteria is not needed for survival of the mutant lacking the methyltransferase. Similar genome-wide analyses can likely to be used to define the cellular roles of many additional uncharacterized DNA methyltransferases.

## Introduction

DNA methylation—the covalent attachment of methyl moieties to specific nucleotides in the genome by DNA methyltransferases (MTases)—is a fundamental mechanism for epigenetic regulation in all domains of life (reviewed in [1,2]). Bacterial DNA MTases principally generate three modified DNA bases [3,4]: 6-methyladenine (6mA), 4-methylcytosine (4mC) and 5-methylcytosine (5mC). Most bacterial MTases are components of restriction-modification (R-M) systems; these MTases modify target DNA sequences in order to protect them from digestion by a cognate restriction enzyme, which is typically co-transcribed. R-M systems enable digestion of horizontally acquired DNA sequences that lack appropriate methylation marks, and thus protect bacteria from selfish elements and phage predation [5]. However, a subset of MTase genes are not accompanied by a cognate restriction enzyme, and a few of these so-called ‘orphan’ MTases are known to regulate diverse host cell processes (reviewed in [2,6,7]). For example, the 6mA MTase Dam, which is found in *E*. *coli* and many other gamma proteobacteria, regulates DNA replication [8], mismatch repair [9], transposition [10] and pilus biogenesis [11], while the adenine MTase CcrM is a critical regulator of the *Caulobacter crescentus* cell cycle [12]. Recently, Dcm, an *E*. *coli* orphan MTase that produces 5mC, was found to modulate antibiotic resistance [13], translation [14], and stationary phase gene expression [15]. Additionally, MTases of Type III R-M systems have also been found to mediate phase variation [16,17]. Nonetheless, roles for the majority of bacterial MTases—which are predicted in over 90% of genomes [18]—have not been defined.

Here, we investigated the importance of DNA methylation in the cholera pathogen, *Vibrio cholerae*. The canonical El Tor O1 *V*. *cholerae* strain N16961 [19] is predicted to encode 4 MTases [18] with distinct catalytic activities. The *V*. *cholerae* Dam homologue has been shown to be critical for replication of one of the organism’s two chromosomes, and is consequently essential for survival [20,21]. In contrast, *vc1769* (*hsdM*), *vca0198 (vchM)* and *vca0447* have been found to be not essential, either through targeted mutagenesis [22] or in transposon insertion sequencing screens [23–25]. VC1769 is a homologue of the *E*. *coli* 6mA-generating HsdM, which is part of a type I R-M system [26], and *vca0447*, a putative orphan adenine MTase, remains uncharacterized to date. VchM is present with almost 100% identity in 91% (10/11) of complete *V*. *cholerae* genome sequences at NCBI, but is absent from more than 90% (20/22) of non-cholerae Vibrios (S1 Fig), and thus appears to have been acquired by horizontal gene transfer. VchM was previously characterized as an orphan 5mC MTase that targets the consensus sequence RCCGGY [22,27], but the importance of this enzyme to host gene expression has not been defined.

Here, we demonstrate that VchM is required for optimal *V*. *cholerae* growth, both *in vitro* and during infection. Bisulfite sequencing defined the *V*. *cholerae* 5-methylcytosine methylome, and RNA sequencing analyses revealed that VchM regulates expression of genes important in a variety of cellular processes, potentially through direct intragenic methylation. Unexpectedly, transposon insertion sequencing-based analyses of *vchM* genetic interactions revealed that deletion of *vchM* suppresses the essentiality of the σ^E^ envelope stress response pathway. Additional transposon mutagenesis studies identified host genes that are required for envelope stability, in whose absence σ^E^ is induced. Many of these genes, especially those involved in the modification of the lipopolysaccharide inner core, contain VchM recognition sites. Mutational analyses suggest that VchM cytosine methylation directly downregulates the expression of some of these LPS modification genes. Thus, our findings show that *V*. *cholerae* has co-opted the horizontally acquired VchM DNA methyltransferase to regulate a diverse array of critical cellular processes.

## Results

### vchM is required for optimal *V*. *cholerae* growth in vitro and during infection

**Fig 1:**
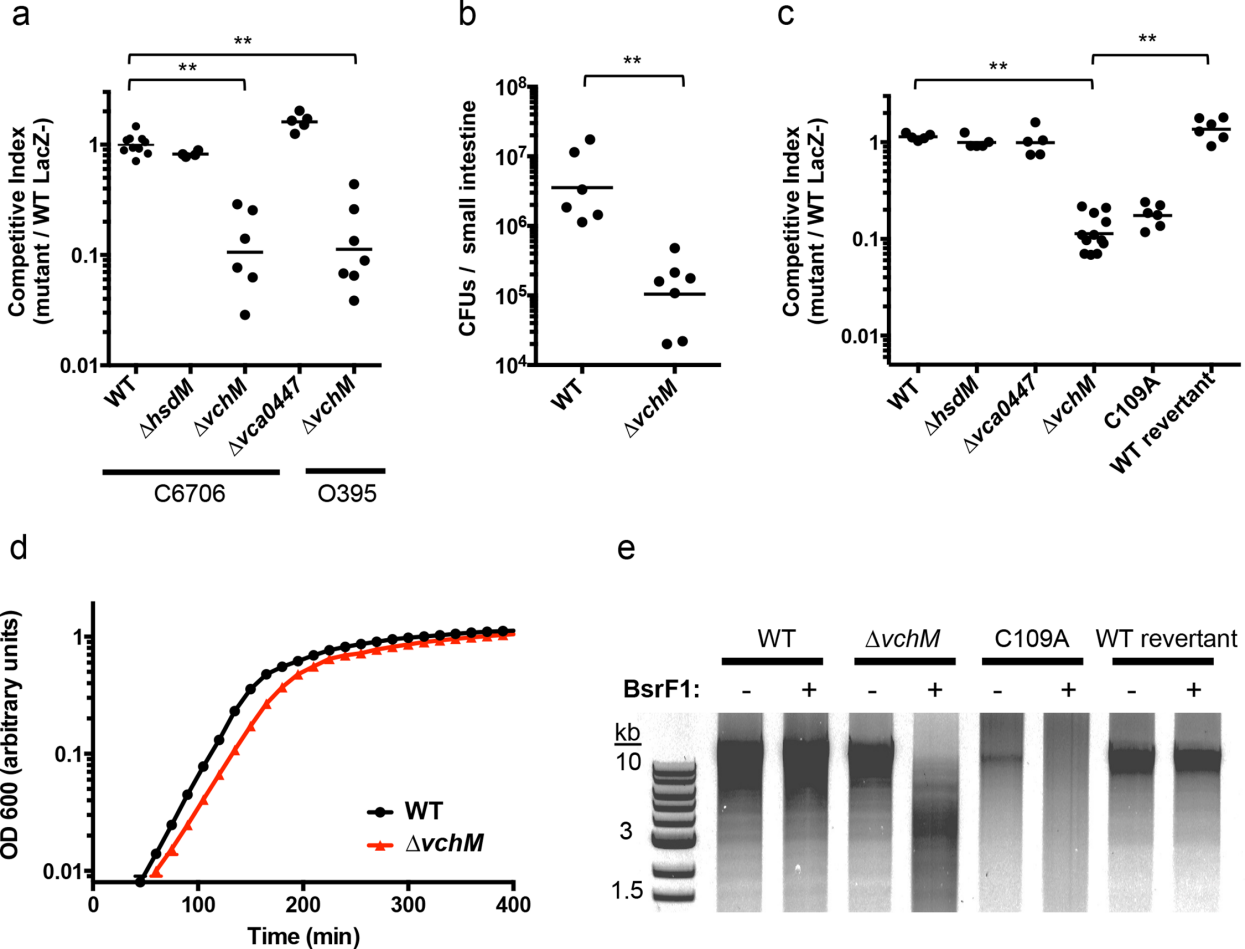
VchM methyltransferase activity promotes *V*. *cholerae* growth and pathogenicity. (A) Mice were co-infected with wildtype *V*. *cholerae* (either C6706 or O395) and a differentially marked methyltransferase mutant, which could be distinguished by blue/white screening. The competitive index was calculated from the ratio of mutant (LacZ^+^) to WT (LacZ^-^) cells recovered after infection divided by the corresponding ratio of the inoculum. WT represents a control competition between LacZ^+^ and LacZ^-^ *V*. *cholerae* C6706. **p-value <0.01. (B) Mice were singly infected either with wt or *∆vchM V*. *cholerae* C6706 and the total number of CFUs recovered per small intestine is shown. **p-value <0.01. (C) Differentially marked strains (LacZ^-^, *∆hsdM*, *∆vca0447*, and either *∆vchM*, a *vchM* active site mutant (C109A), or a deletion mutant into which the wt gene had been reintroduced (wt revertant)) were co-cultured in LB with wt cells and the competitive indices were calculated as in (A). **p-value <0.01. (D) The growth rate of wt and *∆vchM* cells in LB was monitored over time using OD 600 measurements. (E) Genomic DNA from the indicated strains was digested with SalI and the methylation-sensitive enzyme BsrFI, which does not cleave methylated RCCGGY motifs.

We created in-frame deletion mutants of *V*. *cholerae’s* non-essential MTases—*vc1769* (*hsdM*), *vca0198* (*vchM*), and *vca0447*—and compared the growth of each mutant to an isogenic wild type (wt) El Tor biotype strain during infection of suckling mice, using competition assays. The *∆vchM* mutant displayed significantly attenuated (~10 fold) growth *in vivo*, while the recovery of the *∆hsdM* and *∆vca0447* mutants was equivalent to that of WT cells (Fig 1A). When *vchM* was deleted in the prototypical classical biotype *V*. *cholerae* strain, O395, ten-fold attenuation *in vivo* was also observed (Fig 1A). Similarly, infant mice inoculated solely with *∆vchM V*. *cholerae* accumulated 30-fold fewer intestinal bacteria than did animals infected with WT *V*. *cholerae* (Fig 1B). The *∆vchM* mutant was also outcompeted by wt cells when grown *in vitro* (Fig 1C) and displayed a reduced doubling rate when grown in monoculture (Fig 1D). Together, these data indicate that *∆vchM* cells, unlike other mutants lacking an orphan 5mC MTase [15], have an intrinsic growth defect that manifests both during *in vitro* and *in vivo* growth.

To confirm that the growth defect of the *∆vchM* mutant is due to the absence of 5mC methylation, rather than to a second site mutation or a non-enzymatic role of VchM, we re-introduced a wildtype or catalytically inactive *vchM* (C109A) allele into the *∆vchM* mutant at the endogenous locus. Restriction digests confirmed that gDNA from the strain into which the wt sequence was reintroduced—like that of wt *V*. *cholerae—*was resistant to cleavage by BsrFI, which cannot cleave methylated RCCGGY motifs. In contrast, gDNA from *∆vchM* and C109A *V*. *cholerae* was sensitive as expected (Fig 1E). The WT replacement, but not the C109A replacement, also fully complemented the *in vitro* growth defect of the *∆vchM* parent (Fig 1A and 1C). Thus, the catalytic activity of VchM is required for optimal bacterial growth, suggesting 5mC DNA methylation controls processes necessary for optimal *V*. *cholerae* growth.

### The VchM methylome is stable across different growth states

**Fig 2:**
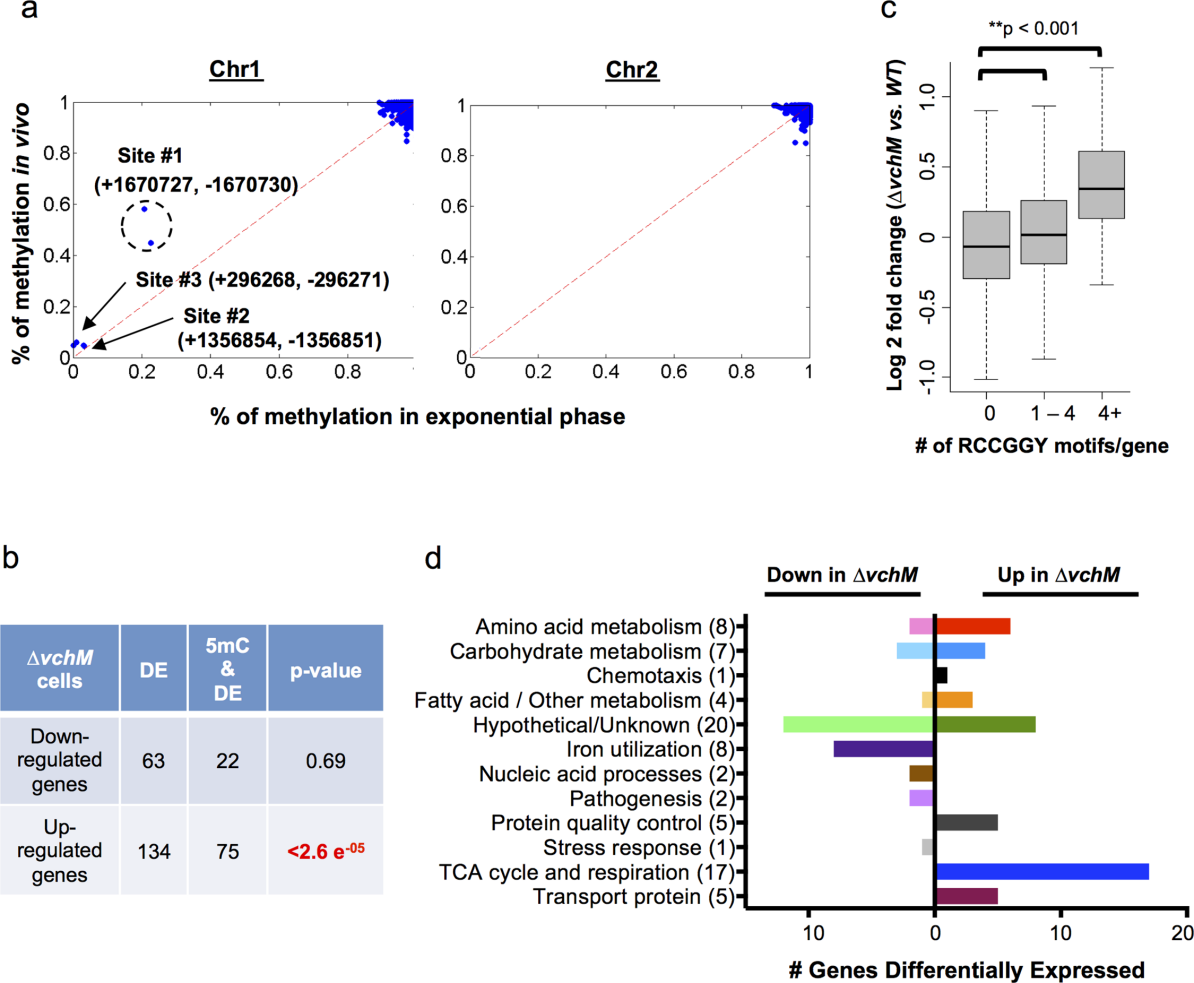
*V*. *cholerae* cytosine methylation and its associations with gene expression. (A) The extent of VchM target site (RCCGGY) methylation on chromosomes I and II, as determined by bisulfite sequencing of DNA from exponential phase cultures, is plotted against the extent of methylation for these sites during infection. Three sites (#1-#3) were found to be persistently non-methylated; numbers designate the genomic position of each site on the forward (+) or reverse (-) strand. (B) Genes that were significantly (p-val <0.01) differentially expressed by wt and *∆vchM* cells were correlated with the presence of intragenic RCCGGY motifs. Significance was assessed using a Fisher’s exact test by the comparing the fraction of differentially expressed genes containing RCCGGY motifs (e.g., 22 out of 63) to the fraction of all genes in *V*. *cholerae* containing RCCGGY motifs (1460 out of 3842 genes). Significant enrichment of motifs was found within only the genes that were upregulated in *∆vchM* cells. DE = number of differentially expressed genes; 5mC = number of genes containing methylated RCCGGY motifs. (C) Differences in transcript abundance between exponential phase cultures of *∆vchM* and WT cells are plotted relative to the number of RCCGGY motifs present within the coding region for each gene. The boxes represent the fold change of genes in the 25%-75% quartile with the median fold change shown as a line. The whiskers represent 1.5 fold of the interquartile range (the third quartile minus the first quartile) away from the box. (D) The numbers of genes that are differentially expressed (p-value <0.05) by *∆vchM* strains of C6706 and O395 *V*. *cholerae* compared to their parental strains are shown for a variety of biological categories.

Previous work revealed that VchM recognizes and methylates a consensus sequence of RCCGGY (methylated residue underlined) [22]. Interestingly, the distribution of RCCGGY motifs is not uniform across the genome (S2 Fig). A previous study evaluated the methylation status of these sites and found that three VchM sites in *V*. *cholerae* are undermethylated in late exponential phase, compared to the rest of the genome [27]. However, it remained unknown whether *V*. *cholerae’s* pattern of 5mC methylation could vary between different growth states, as was observed for the *E*. *coli* orphan 5mC DNA MTase, *dcm* [15]. Thus, we used bisulfite sequencing, in which non-methylated cytosines are converted into uracils and detected as C to T transitions, to assess the methylation status of all cytosines in the genomes of bacteria in exponential and stationary growth phases, as well as *V*. *cholerae* that had been isolated from infected rabbit intestines. This approach was highly specific and sensitive, revealing a clear distinction between methylated cytosines within VchM’s RCCGGY target sites and non-methylated cytosines in other sequence contexts (Fig 2A, S3A Fig).

The bisulfite sequencing results (GEO Accession number GSE73975) revealed that virtually all VchM motifs in the genome are methylated with high frequency in exponential phase, i.e., RCCGGY sites on >90% of the DNA molecules were fully methylated (Fig 2A). Of the ~2100 VchM motifs in the genome, only 3 were persistently non-methylated (methylated <20% of the time on both strands). These sites, which were previously described, are all located within intergenic regions between two divergently oriented genes, and are thought to be non-methylated in exponential phase due to binding by transcription factors [27]. Most VchM sites were also methylated with high frequency in stationary and *in vivo* grown cells (Fig 2A, S3B Fig). The non-methylated sites #2 and #3 were similarly non-methylated during infection, but site #1 (Fig 2A), which is located in between VC1558 and VC1559, showed increased methylation *in vivo* (~51% of cells) compared to *in vitro* exponential grown cells (~20% of cells). While the functional consequence of this difference remains to be defined, our data show that the methylation of VchM sites is highly saturated across the genome (i.e., virtually all RCCGGY motifs are fully methylated) and is not drastically altered during *V*. *cholerae* growth in the intestine.

### VchM methylation correlates with altered gene expression

To investigate the impact of cytosine methylation on *V*. *cholerae* gene expression, we compared the transcriptomes of WT and *∆vchM* cells in the C6706 strain background (GEO Accession number GSE73974). We identified 134 genes with significantly (p-value <0.05) elevated transcript abundance in the *∆vchM* mutant, and 63 genes with reduced transcript abundance (S1 Table). While there was no significant enrichment of VchM motifs within the genes with reduced transcript levels, seventy-five of the genes with elevated transcript levels contained a RCCGGY motif, which is significantly more than would be predicted by chance alone (Fig 2B). The correlation between the presence of VchM target sites and increased transcript abundance in the *∆vchM* mutant was also observed when analysis was not restricted to genes with significantly altered transcript levels. Genome wide, relative transcript levels were significantly higher in *∆vchM* cells versus wt for genes that contained 1 or more VchM targets within 200 bp of their transcriptional start sites [28] (S4A Fig). We also observed a significant association between the number of RCCGGY target sites within a gene’s coding region and its expression change in *∆vchM* cells, especially for genes containing more than 4 target sites (Fig 2C). This result suggests that VchM methylation reduces gene expression of some genes. This significant association was independent of the genes’ GC contents (S4B Fig) and was not observed for several other similar motifs (S4C Fig). Additionally, no strong association between the precise location of RCCGGY sites within genes and differential expression was observed; the motifs are similarly distributed throughout the coding region of all genes as well as those that are differentially expressed (S4D Fig).

To identify pathways that are consistently regulated (directly or indirectly) by VchM, we compared transcriptomic analyses for C6706 and O395 *∆vchM* mutants and the corresponding wt strains. We identified 79 genes that were significantly and differentially (p-value < 0.01) expressed in the absence of VchM in both biotypes (Fig 2D, S2 Table). Approximately 25% of these are hypothetical genes, while the remainders are predicted to participate in a variety of critical processes, including energy production (22%), amino acid metabolism (10%) and iron utilization (10%). Expression of the iron utilization genes was reduced in *∆vchM* cells compared to wt cells, and thus is unlikely to be directly controlled by VchM-dependent methylation. Likewise, while the genes involved in the TCA cycle and respiratory chain are upregulated in *∆vchM* cells, most do not contain RCCGGY motifs, suggesting that their elevated expression may also be an indirect response to the loss of methylation.

### vchM is required to maintain rpoE essentiality

**Fig 3:**
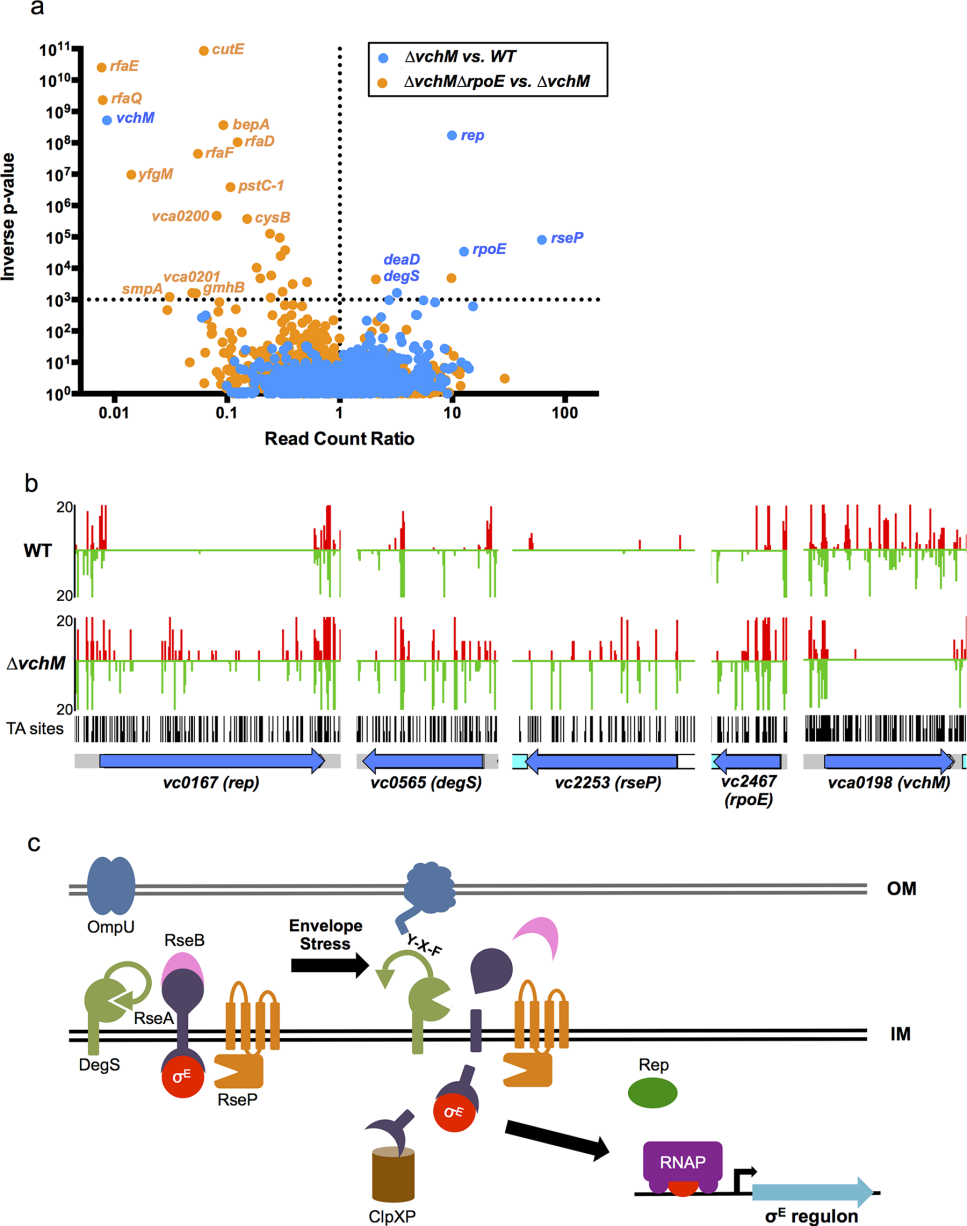
TIS-based identification of loci that interact genetically with *vchM* or *rpoE*. (A) For two comparative analyses (*∆vchM* vs. wt and *∆vchM ∆rpoE vs*. *∆vchM)*, the read count ratio of transposons inserted into each genetic locus was determined, and is plotted against the inverse of the associated p-value as determined by Mann-Whitney U analysis of read counts. Genes plotted above the horizontal line are considered significantly different in each analysis (p-value <0.001). (B) The raw number of reads originating from insertions on the forward (red) or reverse strand (green) in wt and *∆vchM* insertion libraries are shown for selected loci from both libraries. All potential insertion sites (TA dinucleotides) are marked by black bars. (C) The predicted σ^E^ stress response pathway in *V*. *cholerae*.

To gain more insight into the physiology of the *vchM* mutant, in particular the processes that it utilizes for growth, we used a transposon-insertion sequencing (TIS) approach to screen for loci that are differentially required for survival in *∆vchM* or wt *V*. *cholerae*. High-density transposon libraries were created in WT and ∆*vchM* cells grown in rich media, and all the transposon insertion sites in each library were sequenced. No genes specifically required by the *∆vchM* mutant (i.e., genes lacking insertions only in the *∆vchM* background) were identified in this screen other than the positive control, *vchM* itself. However, we unexpectedly identified 5 genes (Table 1, Fig 3A) in which there was a significantly higher frequency of transposon insertion in *∆vchM* cells compared to the wt strain (Fig 3B, S5 Fig), suggesting such genes are potentially more important to the survival or optimal *in vitro* growth of the wt strain than that of the *∆vchM* mutant. Remarkably, four of the five genes—*rseP*, *degS*, *rpoE* and *rep*—are known to participate in the σ^E^ envelope stress response pathway (reviewed in [29]).

**Table 1:** *vchM* genetic interactions identified by transposon-insertion sequencing.

Locus	Name	Average p-value in all MWU tests	Average Read Count Ratio (∆*vchM* / wt)	Predicted Function
*vca0198*	*vchM*	1.91E-09	0.01	5mC DNA methyltransferase
*vc0167*	*rep*	5.83E-09	9.91	ATP-dependent DNA helicase
*vc2253*	*rseP*	1.24E-05	62.22	Second protease that cleaves the RseA antisigma factor
*vc2467*	*rpoE*	2.94E-05	12.66	Envelope stress responsive sigma factor
*vca0804*	*deaD*	6.10E-04	3.21	Putative ATP-dependent RNA helicase
*vc0565*	*degS*	0.001	2.74	First protease that cleaves the RseA antisigma factor

In the σ^E^ stress response pathway, which has been best described in *E*. *coli* but has also been characterized in *V*. *cholerae* (Fig 3C), σ^E^ is sequestered at the membrane by an integral membrane anti-sigma factor, RseA [30–32]. Upon envelope stress, specific outer membrane proteins become unfolded and expose a terminal YXF motif, which interacts with and activates the intramembrane protease DegS [33,34]. Envelope stress may also dysregulate outer membrane biosynthesis, leading to increased periplasmic LPS, which binds and inactivates an additional DegS inhibitory factor, RseB [35,36\. The presence of LPS and OMP stimuli permits DegS to cleave within the periplasmic domain of RseA [34,37,38], allowing a second intramembrane protease, RseP, to cleave the cytosolic domain of RseA [38–40]. These proteolytic cleavages lead to the release of σ^E^ into the cytosol, where it can engage RNA polymerase to direct transcription of the σ^E^ regulon, which reduces *de novo* expression of outer membrane proteins while facilitating the refolding of existing ones [41–44]. An ATP-dependent helicase, *rep*, was also recently found to be required for induction of some σ^E^ regulon genes [45], though the exact mechanism by which this occurs is unknown.

Both in *E*. *coli* and *V*. *cholera*e, *rpoE* and related factors have been found to be essential [46–49], although suppressor mutations that permit survival of *rpoE*-deficient cells have also been identified [47,50–52]. In *V*. *cholerae*, the outer membrane protein OmpU is the predominant determinant of *rpoE* activation, both under normal growth conditions and in response to envelope stress [53], and *ompU* mutations are the most frequently identified suppressors of *rpoE* essentiality [47]. Suppressors directly associated with LPS synthesis or processing have not been identified in *V*. *cholerae*.

**Fig 4:**
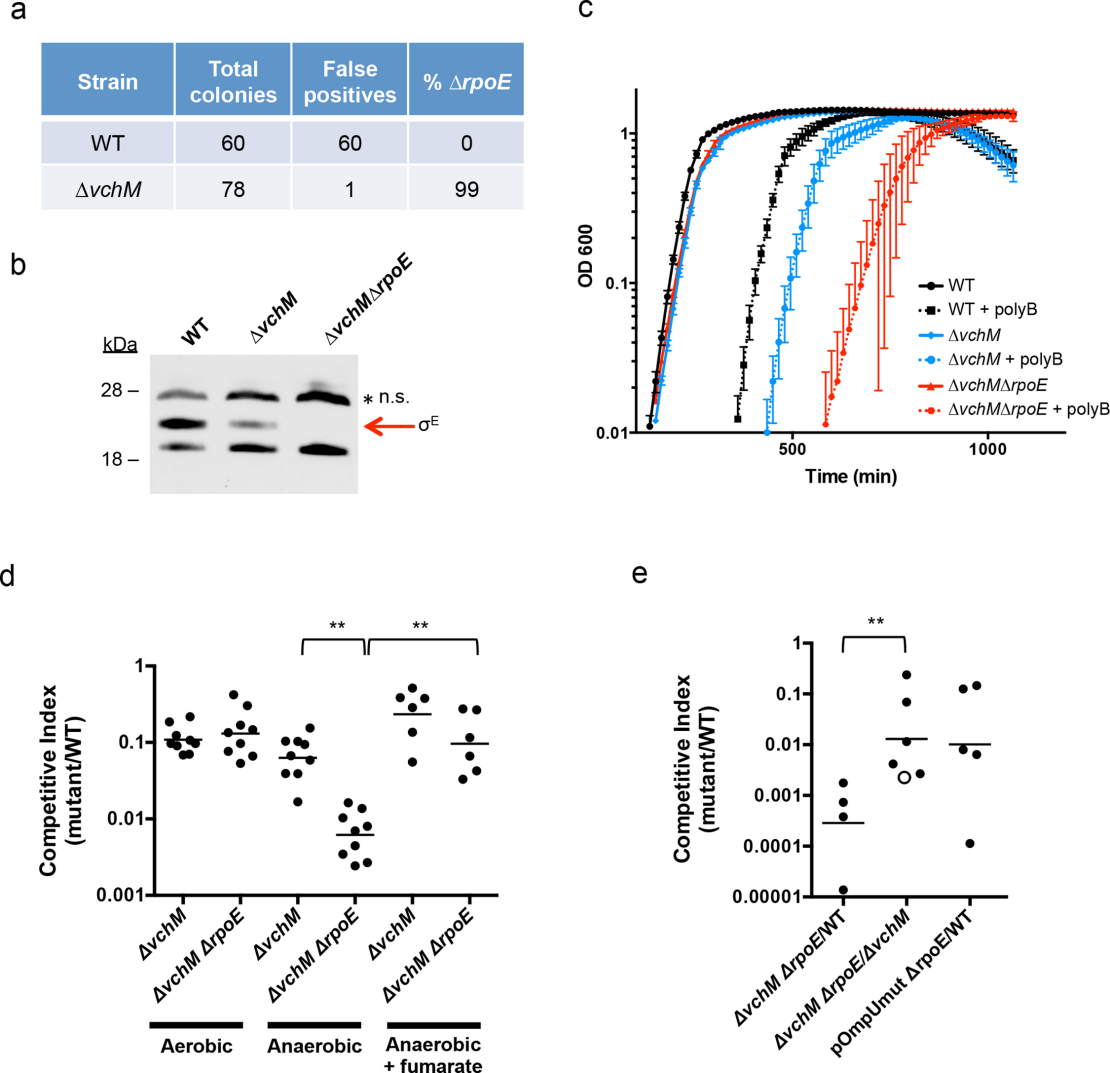
Deletion of *vchM* suppresses the essentiality of *rpoE* but *∆vchM ∆rpoE* mutants show impaired growth under stress conditions. (A) Allelic exchange to replace *rpoE* with a kanamycin resistance cassette was performed in WT and *∆vchM* cells. After patching colonies to determine if candidate colonies still retained the knockout vector, the false positive and *rpoE* deletion frequencies were calculated. (B) A representative western blot of lysates from cultures of WT, *∆vchM* and *∆vchM ∆rpoE* cells probed with polyclonal antisera against σ^E^. n.s. = non-specific signal. (C) Culture density (OD 600) of the indicated strains was monitored for cells grown in LB and in LB supplemented with polymyxin B (poly B). (D) *In vitro* competition assays were performed between LacZ- *V*. *cholerae* and the indicated mutants. Cultures were grown in LB under aerobic or anaerobic conditions and supplemented with fumarate as noted. Competitive indices were calculated as in Fig 1. (E) Competitive infections were performed using differentially marked versions (LacZ^+/-^) of the indicated strains. Competitive indices were calculated for bacteria in intestinal homogenates as in Fig 1A. The open circle represents the limit of detection where no *∆vchM ∆rpoE* mutants were recovered.

Our TIS analysis revealed that *rseP*, *degS*, *rpoE* and *rep*, which are all required for activation of the *rpoE* pathway, appeared dispensable in *∆vchM* cells, suggesting that disruption of *vchM* may be a previously unrecognized suppressor of *rpoE* essentiality in *V*. *cholerae*. To validate this hypothesis, we attempted to delete *rpoE* in both WT and *∆vchM* cells, using a targeting construct designed to replace *rpoE* with a kanamycin-resistance cassette. As in previous analyses [47], a very high percentage (here, 100%) of the putative *∆rpoE*::aphR mutants generated in the wt background were false positives that retained an additional antibiotic resistance marker, reflecting retention of the targeting vector and failure to disrupt *rpoE* (Fig 4A). In contrast, 99% of *∆vchM ∆rpoE* candidates did not retain the allelic exchange vector, suggesting that deletion of *rpoE* in the *∆vchM* strain can be readily achieved. Furthermore, a putative *∆vchM ∆rpoE* colony was randomly selected and the absence of σ^E^ was confirmed by western blot analysis (Fig 4B). Thus, *V*. *cholerae* does not require the presence of *rpoE* in the absence of *vchM*.

Interestingly, our western blots also revealed that basal levels of σ^E^ were only ~30% of WT levels in the *∆vchM* mutant (Fig 4B). Since σ^E^ expression and activity is elevated in response to envelope stress, the lower basal level σ^E^ in *∆vchM* cells suggests that 5mC methylation by VchM may contribute to envelope stress, and thereby modulate production of σ^E^. Reduced activation of σ^E^ in cells lacking VchM could allow *rpoE* to be dispensable in these cells, at least under the conditions at which the mutants were selected.

### rpoE is required for growth under stress conditions and during infection

In addition to confirming the viability of *V*. *cholerae* lacking *vchM* and *rpoE* under relatively favorable growth conditions (rich media), we assessed whether these mutations altered bacterial growth in a variety of more challenging environments. We found that growth of the *∆vchM ∆rpoE* mutant was equivalent to that of the *∆vchM* parent strain in LB monocultures (Fig 4C), and that it exhibited an equivalent ~10-fold growth impairment as *∆vchM* cells in competition assays with WT cells in aerobic LB culture (Fig 4D). However, in the presence of the outer membrane targeting antimicrobial peptide, polymyxin B, which is known to induce σ^E^ above baseline levels in *V*. *cholerae* [53], growth of the *∆vchM ∆rpoE* mutant was reduced far more than that of the *∆vchM* strain (Fig 4C). Thus, the strain that has no capacity to activate the σ^E^ stress response pathway (e.g., *∆rpoE*) is at a marked growth disadvantage in envelope stress-inducing environments, and the *∆vchM* mutation does not suppress *V*. *cholerae’s* need for *rpoE* under these conditions.

Similarly, deletion of *vchM* did not suppress *V*. *cholerae’s* need for σ^E^ during growth in the infant mouse intestine. In *in vivo* competition experiments, the *∆vchM ∆rpoE* mutant was recovered at more than a 1000-fold reduced frequency (CI ~0.0007) than the wt strain from the infant mouse intestine (Fig 4E), and with 100-fold reduced frequency when competed against the *∆vchM* parent strain (CI ~0.01). These results suggest that deletion of *rpoE* exacerbates the ~10-fold *in vivo* proliferation deficiency of the ∆*vchM* mutant, which has a CI vs the wt strain of ~0.1, by a factor of 100. A similar ~100-fold effect of *rpoE* disruption on *V*. *cholerae* growth *in vivo* was observed using an *rpoE-*deficient strain containing an *ompU* promoter mutation (pOmpU mut) that reduces porin expression. Since previous studies showed that deletion of *ompU* does not impair *V*. *cholera*e intestinal colonization [54], the *in vivo* attenuation of this double mutant can be fully explained by the absence of *rpoE*.

Finally, since the RNA-seq results revealed that *∆vchM* cells had elevated expression of TCA cycle and respiration genes, we assessed the growth of the *∆vchM* and *∆vchM ∆rpoE* strains in anaerobic conditions, which would also be encountered during host infection. Relative to the WT strain, the *∆vchM* mutant exhibited a similar growth defect in both aerobic and anaerobic conditions (Fig 4D). In contrast, the *∆vchM ∆rpoE* mutant was significantly more attenuated than the *∆vchM* parent during anaerobic growth, raising the possibility that the *in vivo* attenuation of *∆rpoE* strains is in part explainable by the anaerobic conditions in the intestine (Fig 4D). Interestingly, the deleterious effect of the anaerobic environment on the *∆vchM ∆rpoE* mutant was eliminated by the addition of fumarate, which acts as a terminal electron acceptor for *V*. *cholerae* respiration during anaerobic growth [55]. Thus, non-respiratory growth (e.g., fermentation) is specifically deleterious for *V*. *cholerae* lacking *rpoE*. It is possible that a fermentative byproduct is selectively toxic to *∆rpoE* cells, or that physiological changes linked to fermentative growth lead to increased cell envelope stress.

### TIS identifies genes required for envelope stability

**Fig 5:**
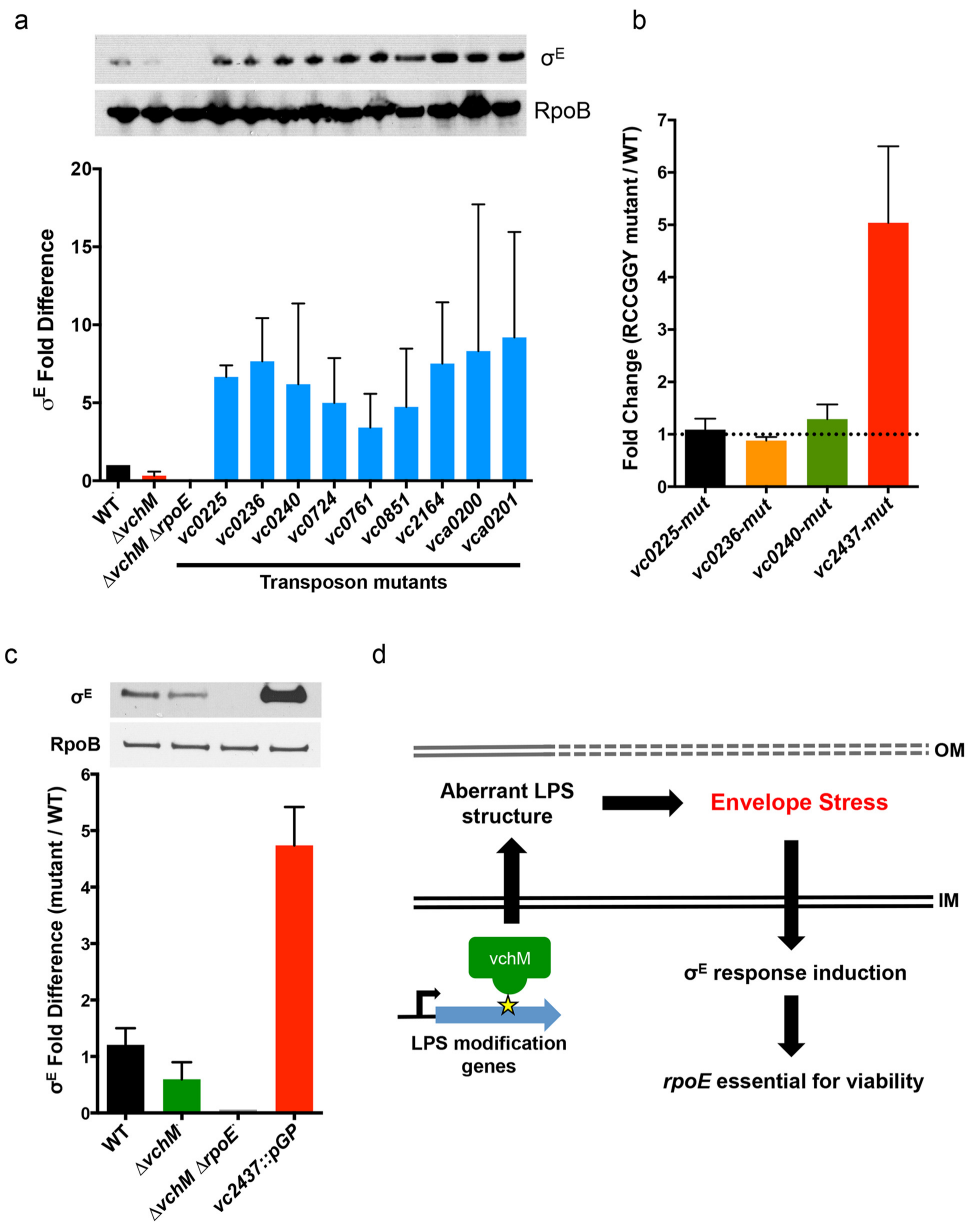
Other cell envelope-related loci and VchM control of LPS modification contribute to basal levels of σ^E^. (A) Western blotting and relative abundance (compared to wt) of σ^E^ in mutants lacking genes that could not be disrupted in the *∆vchM ∆rpoE* mutant. Mutants contain transposon insertions in the genes of interest, and are derived from a wt strain, not *∆vchM*. RpoB was used as a loading control to normalize for total protein. Results are the average of three independent experiments. (B) Relative abundance of transcripts for LPS synthesis genes in mutants containing synonymous mutations that abolished RCCGGY motifs in these genes. Abundance is relative to transcript levels in wt cells, as determined by qRT-PCR in three independent biological replicates. (C) The relative abundance of σ^E^ in wt, *∆vchM* and *∆vchM ∆rpoE* cells and in a *vc2437* insertion mutant (*vc2437*::pGP). Error bars represent data from two independent replicates normalized to wt basal σ^E^ levels. (D) Model for VchM-mediated modulation of the σ^E^ envelope stress response in *V*. *cholerae*. In wt cells, VchM methylation of some LPS modification genes restricts their expression, leading to aberrant LPS structure, generation of envelope stress and the induction of the σ^E^ response, which renders *rpoE* essential.

We used transposon-insertion sequencing to begin to explain why the presence of VchM correlates with higher basal σ^E^ levels. We hypothesized that VchM regulates genes whose products are necessary for optimal envelope stability and conducted a screen to identify genes whose absence leads to increased envelope stress. In cells with a functional envelope stress pathway, disruption of genes that are required for envelope stability should induce envelope stress that can be ameliorated by the σ^E^ pathway. However, in cells lacking *rpoE*, such mutations (and the resulting stress) may be lethal. Using TIS, we identified 13 candidate genes that could tolerate transposon insertion in the *∆vchM* background but not in *∆vchM ∆rpoE* cells (Fig 3A, Table 2). Many of these genes are known to be involved in envelope biogenesis (Table 2), including several genes required for modifying the inner core component of LPS (orange shaded genes). Consistent with our hypothesis, disruption of many of these LPS synthesis genes (including *waaC* and *rfaF*) is known to cause induction of the σ^E^ stress response in *E*. *coli* [36,56]. We also identified non-LPS outer membrane-related genes, such as *smpA*, a known σ^E^-regulating gene that is required for assembly of outer membrane beta-barrel proteins (OMPs) and for correct outer membrane biogenesis, and *lnt*, which acylates lipoproteins transported by the Lol complex [57,58,59]. It is known that overexpression of the major outer membrane protein, Lpp, in *E*. *coli*, induces the σ^E^ stress response, potentially by dysregulating outer membrane lipoprotein insertion through the LolA/B complex [44]. Additionally, the screen yielded genes that are not clearly tied to the *rpoE* pathway or outer membrane biogenesis, including *cysB*, a transcriptional activator that regulates sulfur transport [60], and *vca0200* and *vca0201*, two hypothetical genes that are specific to *V*. *cholerae* and lie downstream of *vchM*. Using mutants from an ordered *V*. *cholerae* transposon library [24], we validated our hypothesis that these mutants contain more σ^E^ than wt cells (Fig 5A), suggesting envelope stress is increased in the absence of these genes.

**Table 2:** TIS identification of genes necessary for envelope stability. Genes are grouped based on functioning in LPS biosynthesis, localization to the cell envelope, or an unspecified contribution towards cell envelope processes.

Locus	Name	Average p-value across all MWU tests	Average read count ratio (*∆vchM∆rpoE/ ∆vchM*)	Number of RCCGGY motifs	Predicted Function
LPS Biosynthesis					
*vc0225*	*rfaQ*, *waaC*	4.36E-10	0.01	1	LPS heptosyltransferase; modifies KDO inner core component of LPS
*vc0236*	*rfaF*	2.24E-08	0.06	1	ADP-heptose-LPS heptosyltransferase II; modified KDO component of LPS
*vc0240*	*rfaD*, *htrM*	9.56–09	0.12	1	ADP-L-glycero-D-mannoheptose-6-epimerase (inner LPS core synthesis)
*vc0908*	*gmhB*	6.31E-4	0.05	0	D,D-heptose 1,7-bisphosphate phosphatase (inner LPS core synthesis)
*vc2437*	*rfaE*, *waaE*	3.96E-11	0.01	3	Bifunctional heptose 7-phosphate kinase and heptose 1-phosphate adenyltransferase (LPS inner core synthesis)
Localized to the cell envelope					
*vc0724*	*pstC-1*	2.60E-07	0.11	0	Phosphate ABC transporter
*vc0761*	*yfgM*	1.04E-07	0.01	0	SecYEG translocon accessory protein; anti-RcsB factor (capsule biosynthesis)
*vc0851*	*smpA*	8.18E-04	0.03	0	Small protein A, *rpoE* regulator, outer membrane lipoprotein
*vc0958*	*cutE*,*lntA*	1.17E-11	0.06	0	Apolipoprotein N-acyltransferase lnt; acylates lipoproteins for Lol complex recognition and translocation to outer membrane
*vc2164*	*bepA*	2.72–09	0.09	1	Periplasmic metalloprotease and chaperone; outer membrane maintenance
Unspecified					
*vc1907*	*cysB*	2.69E-06	0.15	0	Sulfur transport transcriptional activator
*vca0200*		2.13–06	0.08	0	Hypothetical
*vca0201*		6.09E-04	0.05	0	Hypothetical

We speculated that expression of some of these loci might be directly regulated by VchM-dependent methylation, which could then allow them to modulate cellular levels of σ^E^. Interestingly, the LPS-related candidate genes are enriched for RCCGGY motifs compared to candidate genes of the other classes (p-value < 0.05 by Fisher’s exact test), so we focused on this subset. We used site directed mutagenesis to replace the RCCGGY motifs in *vc0225*, *vc0236*, *vc0240* and *vc2437* with non-consensus sites that do not alter the protein sequence. While there were no significant differences in mRNA expression of *vc0225*, *0236* and *0240* after RCCGGY ablation, replacing all three RCCGGY motifs in *vc2437* caused an approximately 5-fold increase in transcript abundance compared to WT levels (Fig 5B), suggesting that methylation of *vc2437* by VchM reduces its expression. Consistent with the idea that regulation is methylation-dependent, there was a less than two-fold difference between expression of the wt and mutated locus in the *vchM* background. Furthermore, a mutant harboring a disruption of *vc2437* (*vc2437*::pGP) had significantly elevated basal σ^E^ levels (Fig 5C), suggesting that downregulation of this gene’s expression can induce *V*. *cholerae* envelope stress.

## Discussion

We carried out methylomic, transcriptomic, and genetic analyses of the role of the orphan cytosine MTase, VchM, and uncovered an unexpected connection between cytosine modification and outer membrane stress in *V*. *cholerae*. To our knowledge, VchM is only the second orphan 5mC-catalyzing bacterial MTase to be characterized in depth and genetic interactions comprehensively identified. Strains lacking VchM exhibit impaired growth *in vivo* and during both aerobic and anaerobic growth *in vitro*, and transcriptomic analysis indicates that many important metabolic pathways are altered in *∆vchM* cells. Comparative transposon insertion sequencing analyses of wt and *∆vchM V*. *cholerae* revealed that deletion of *vchM*, suppresses *V*. *cholerae’s* requirement for the σ^E^ envelope stress response pathway, which likely reflects reduced basal activity of this pathway in the *∆vchM* strain. Notably, deletion of *vchM* does not mitigate *V*. *cholerae’s* need for *rpoE* under challenging growth conditions, and by studying the *∆vchM ∆rpoE* mutant we were able to quantify the contribution of σ^E^ during host infection and during anaerobic growth as well as screen for genes that cannot be disrupted in the absence of the σ^E^ stress pathway. These genes, which are likely required for optimal envelope stability, include loci that catalyze the heptose modification of LPS and are enriched for VchM recognition motifs. Our data indicate that methylation directly regulates expression of at least some of these genes, and thus reveals one of the mechanisms by which *vchM* influences *V*. *cholerae* physiology.

Unexpectedly, we found that VchM is required for optimal *V*. *cholerae* growth in standard laboratory media (LB). As deletion of *rpoE* does not further impair growth of the *∆vchM* mutant under this condition, it is unlikely that the mutant’s dysregulation of the σ^E^ envelope stress response pathway alone accounts for its slower proliferation. Instead, additional processes, such as those shown by transcriptomic analyses to be altered by the absence of VchM, (e.g., the amino acid and carbohydrate metabolism pathways and those mediating aerobic respiration), may account for the growth phenotype. However, it remains unclear whether the growth deficiency is due to altered expression of a single pathway or to the cumulative effect of simultaneously dysregulating numerous genetic loci. Nonetheless, our data suggests that the horizontally acquired VchM has become an integral component of *V*. *cholerae’s* regulatory networks.

In wt *V*. *cholerae*, *rpoE* is an essential gene [47]; thus, it is noteworthy that *rpoE* can be deleted in the *∆vchM* strain, as relatively few suppressors of *rpoE* essentiality have been identified. Additionally, as noted above, our analyses indicate that growth of the *∆vchM* and *∆vchM ∆rpoE* strains can be equivalent. Deletion of *vchM* appears to reduce basal expression/activation of σ^E^, likely reflecting a reduced need for this factor under conditions that apparently do not produce significant envelope stress. A similar reduction has been observed in strains in which OmpU levels are reduced and *rpoE* is not essential [47]. However, we do not observe reduced expression of OmpU in the *∆vchM* cells (S6 Fig), suggesting ∆vchM suppresses σ^E^ essentiality through a novel mechanism. Furthermore, deletion of *vchM* does not render *rpoE* dispensable under all growth conditions. We observed marked differences (e.g., 10–100 fold) between the growth of *∆vchM* and *∆vchM ∆rpoE* strains in the presence of an antimicrobial compound that activates the σ^E^ pathway, in an animal model of infection, and during anaerobic growth. These data suggest that the *vchM* mutant retains the ability to activate the σ^E^ regulon in response to envelope (or potentially other) stress, despite its lower basal level of σ^E^.

We performed a TIS screen to identify mutations that are detrimental specifically in the absence of *rpoE*, as such mutations are likely to activate σ^E^, and thus might also be regulated through VchM methylation. This screen identified a variety of loci whose products are associated with the cell envelope, including several factors required for heptose modification of the LPS inner core. As anticipated, mutations in these loci were associated with increased σ^E^ abundance, suggesting these mutants have defects in cell envelope integrity. The deletion of genes involved in LPS modification, especially those catalyzing inner core and Lipid A synthesis, have been previously linked to abnormal LPS structure and σ^E^ activation in *E*. *coli* [36,56,61]. Intriguingly, many genes encoding the LPS modifying enzymes identified by our TIS screen contained RCCGGY motifs, raising the possibility that some of these genes might be directly regulated by VchM methylation. Indeed, synonymous disruption of three VchM targets sites in one locus, *vc2437*, led to enhanced expression of this gene, suggesting that VchM-dependent methylation may limit its expression. Since disruption of *vc2437* leads to increased accumulation of σ^E^, we hypothesize that increased levels of Vc2473 in the *vchM* mutant will reduce stimuli activating the σ^E^ stress response, as outlined in our model (Fig 5D). In *E*. *coli*, two signals are required for RseA cleavage and thus σ^E^ induction: activation of DegS through interaction with unfolded outer membrane proteins [33,34] and displacement of RseB from RseA through titration by periplasmic LPS [36]. Lowered expression of *vc2437* could induce and sustain both of these signals. Reduced heptose modification may produce aberrant periplasmic LPS molecules that bind and compete RseB away from RseA, lowering the threshold for DegS activation [35], while simultaneously, aberrant LPS may alter outer membrane structure, facilitating the misincorporation and unfolding of outer membrane proteins.

Additional VchM targets may also exhibit methylation-regulated expression, since transcriptomic and genomic analyses showed that there is a significant correlation between the presence of intragenic RCCGGY motifs and increased expression of these genes in *∆vchM* cells. However, it is likely that cytosine methylation does not universally modulate gene expression in *V*. *cholerae*, since mutation of the single RCCGGY motif in each of three LPS modification genes did not detectably alter gene expression. Genes containing 5 or more motifs showed the greatest elevation in expression in the *vchM* mutant, raising the possibilities that the effects of methylation may be additive within a single gene and/or that a threshold level of methylation must be present for regulation to occur. It is likely that additional regulatory factors also constrain the influence of methylated bases on gene expression.

VchM is the second bacterial orphan 5mC-catalyzing MTase to be characterized extensively at the genomic and transcriptomic levels, and the first for which genetic interactions have been comprehensively defined. Interestingly, our findings reveal striking differences between the functional consequences of methylation by VchM and the previously characterized *E*. *coli* 5mC-catalyzing orphan MTase, Dcm [15]. While Dcm regulates stationary phase expression of ribosomal proteins and mediates resistance to antibiotics in *E*. *coli* [13–15], VchM is required for optimal bacterial growth and modulates cell envelope stress responses in *V*. *cholerae*. At the genomic level, the *E*. *coli* genome is enriched in Dcm targets (CCWGG) [15], while the VchM target, RCCGGY, is not overrepresented in the *V*. *cholerae* genome. Furthermore, Dcm sites are undermethylated during exponential phase and rise during entry into stationary phase, while VchM target sites are nearly fully methylated under all growth states tested, including during infection, suggesting that the two enzymes differ in their expression and/or activity. While increases in gene expression were observed in both ∆*dcm* and *∆vchM* cells, differentially expressed genes in *∆dcm* strains are not enriched for intragenic CCWGG motifs and are instead thought to be controlled indirectly through *rpoS*-dependent regulation [14,15]. In contrast, there is a significant association between the presence of intragenic VchM targets and elevated gene expression in *∆vchM* cells, and mutational analyses suggest that the methylation of these motifs can dampen gene expression. Thus, despite sharing similar catalytic activities, different orphan 5mC MTases can regulate diverse processes through different mechanisms.

The mechanism(s) by which methylation alters gene expression have not been characterized, but many possibilities can be envisioned. For example, methylation within transcribed sequences may influence transcriptional attenuation or transcript half life, perhaps due to effects upon transcript or template structure. Methylation might also influence transcription initiation, e.g. by influencing binding of regulatory factors that control gene expression. It should also be noted that many genes whose expression differs between wt and *vchM V*. *cholerae* are likely indirectly controlled by methylation, e.g., are governed by factors influenced by methylation, but are not themselves methylated. Furthermore, methylation may globally alter chromatin structure in ways that modulate gene expression. Investigation of the precise means by which cytosine methylation in *V*. *cholerae* influences gene expression will be the focus of future studies.

Banerjee et al. [22] found that the closest VchM homologues lie in non-*Vibrio* species, suggesting VchM was horizontally acquired. The introduction of a MTase such as VchM, which modifies thousands of sites and potentially alters gene expression at numerous loci, would exert selective pressure on MTase recognition sites. Consistent with this theory, we found that RCCGGY motifs in *V*. *cholerae* are not randomly distributed; instead, the genome includes regions that are enriched or depleted for VchM recognition sites (S2B Fig). Interestingly, many of the σ^E^-related genes that become dispensable in *V*. *cholerae* lacking *vchM* (as well as *vchM* itself) are located in regions containing a disproportionately low number of RCCGGY motifs (binomial test p-value <4.8e-6; S2B and S2C Fig). It is possible that target sites in these loci have been selected against, as methylation might interfere with beneficial regulatory processes that promote σ^E^ expression.

In conclusion, we found that VchM, a 5mC-catalyzing DNA methyltransferase, serves critical roles in *V*. *cholerae* growth and envelope stress signaling. The processes and mechanisms through which VchM exerts control are strikingly different from *E*. *coli* Dcm, the other well-characterized bacterial 5mC DNA MTase. Thus, future investigations of the regulatory roles of additional bacterial DNA MTases will likely reveal new regulatory schemes for the control of diverse bacterial processes.

## Materials and Methods

### Ethics statement

All animal infections were performed in strict accordance with the recommendations in the Guide for the Care and Use of Laboratory Animals of the National Institutes of Health. All animal protocols were reviewed and approved by the Harvard Medical Area Standing Committee on Animals (protocol 04316). Isofluorane was used for anesthesia.

### Strains, media and culture conditions

All strains were grown on LB Miller (1% NaCl) unless otherwise noted. Antibiotic concentrations used were 200 μg/mL streptomycin (Sm), 50 μg/mL kanamycin (Km), 100 μg/mL ampicillin (Amp), and 50 μg/mL polymyxin B. Wildtype *V*. *cholerae* C6706, *V*. *cholerae* O395 and *E*. *coli* SM10 (lambda *pir*) carrying the Himar1 suicide transposon vector pSC189 [62] were grown at 37°C in LB + Sm and LB + Amp, respectively. Individual transposon mutants from the ordered *V*. *cholerae* transposon library [63] (which contain intact *vchM*) were grown overnight in LB + Sm + Km, or plated as lawns on LB + Sm + Km at 37°C.

### Construction of *V*. *cholerae* deletion, revertant and RCCGGY mutant strains

All primers used for mutant generation can be found in S6 Table. Deletion plasmids for *hsdM* (*vc1769*), *vchM* (*vca0198*) and *vca0447* were derived from the allelic exchange vector pCVD442 [64] using isothermal assembly [65]. Each deletion construct encodes the first five and last four amino acids of the gene of interest, with intervening sequences removed. Suicide plasmids were conjugated into *V*. *cholerae* and sucrose-based counter-selection performed as previously described [66] to create in-frame deletions. Allelic exchange of *rpoE* for a kanamycin resistance gene was carried out using a previously generated suicide vector [47] and a similar protocol as above. However, cells were plated on both 10% sucrose and kanamycin to select for the ∆*rpoE* mutant. For the introduction of a *vchM* C109A mutation into its endogenous locus, an allelic exchange vector containing the entire *vchM* gene with a C109A mutation flanked by 500 bp of upstream and downstream sequence was created, conjugated into *∆vchM* cells, and counter-selected on sucrose plates. Subsequently, a second exchange vector was constructed containing wildtype *vchM* sequence that included 200 bp flanking sequence on each side of the C109 codon, which was used to revert the *vchM* C109A allele back to wildtype.

Similar constructs and methodology were used to replace the single RCCGGY motifs of *vc0225*, *vc0236*, *vc0240* with ACAGGT, ACAGGC, and GCAGGT, respectively. For *vc2437*, a 850 bp fragment encompassing mutations in the three RCCGGY motifs (converted to GCCTGC, GCAGGT and GCAGGC) were synthesized (Integrated DNA Technologies) and used to carry out allelic exchange. For *vc2437* disruption, an internal 850 bp fragment of *vc2437* (derived from the PCR product using primers vc2437-mut1F and vc2437-mut3R) was blunt ligated into the suicide vector pGP704 [67] and conjugated into *V*. *cholerae*. All deletions, reversions and mutations were confirmed by Sanger sequencing (Genewiz).

### Restriction digests

1 ug of purified genomic DNA from the indicated strains was subjected to digestion with SalI in the presence or absence of BsrFI at 37°C for 30 minutes. The reactions were separated by gel electrophoresis, stained with ethidium bromide and imaged on a FujiFilm FLA-5100 fluorescent imager.

### Growth curves, *in vitro* and *in vivo* competition experiments

For growth curves, overnight *V*. *cholerae* cultures were diluted 1:1000 in LB + Sm and any additional chemicals as indicated. These cultures were grown at 37°C in a Bioscreen C optical density reader (Growth Curves USA) with OD600 measurements taken at 15 minute intervals for 12 hours. For competitive growth experiments, overnight stationary cultures of wildtype and mutant *V*. *cholerae*, one of which was *lacZ-*, were independently diluted 1:1000 in LB + Sm and then mixed in a 1:1 ratio. 20 μl of the diluted mixture was inoculated into 2 mL of LB + Sm and grown at 30°C for 24 hours. At the start and end of the experiment, cells were diluted and plated onto LB + 60 μg/mL X-gal to enumerate the ratio between wildtype and mutant cells. For anaerobic experiments, overnight cultures of *V*. *cholerae* were diluted into pre-warmed LB + Sm that was pre-depleted for oxygen by sitting overnight anaerobically at 37°C. Diluted cells were mixed and inoculated into 2 mL of oxygen-depleted LB + Sm (+ 50 μg/mL fumarate when indicated) and after 24 hours of growth at 37°C, cultures were removed from anaerobic conditions and the competitive index determined on X-gal as above.

For *in vivo* competitions, overnight stationary cultures of LacZ- wildtype or mutant *V*. *cholerae* were diluted and mixed 1:1 as above. 50 uL of the diluted culture was orally inoculated into 5-day-old suckling mice (Charles River). After 24 hrs, the mice were sacrificed, the small intestine homogenized using a mini-beadbeater-16 and two 3.2mm stainless steel beads (BioSpec Products Inc., Bartlesville, OK, USA) for 2 minutes, and dilutions of the homogenate were plated on LB + Sm + 60 ug/mL X-gal plates to enumerate the ratio of wildtype and mutant bacteria.

### Bisulfite sequencing

Genomic DNA was extracted from two biological replicates of exponential phase, overnight stationary phase and frozen rabbit cecal fluid *V*. *cholerae* using the Wizard Genomic DNA purification kit (Promega). Bisulfite conversion of the DNA was carried using the EZ DNA Methylation Kit (Zymo) twice to ensure high conversion efficiency. The converted DNA was then amplified by PCR using the Kapa HiFi Uracil+ polymerase for 12–15 cycles (Kapa biosystems) and sequenced on the MiSeq platform (Illumina). Bismark [68] was used to call 5mC sites from the bisulfite sequencing data. Each cytosine site was assigned with two counts, representing the numbers of reads that had a C->T conversion (non-methylated) and those that did not have a C->T conversion (methylated). The fraction of methylation was then calculated for each cytosine site and a minimum total coverage of 10x was used to filter out cytosine sites with too few read counts for estimating the methylation frequency.

### RNA sequencing

Purified mRNA was extracted from two biological replicates of exponential phase *V*. *cholerae* and converted to cDNA as previously described [69]. RNA sequencing was performed on a HiSeq 2500 with 100bp single-end reads. To call differentially expressed genes from RNAseq data, we first mapped raw RNA reads for each sample to the Genbank *V*. *cholerae* El Tor N16961 reference (Accession number: NC_002505 for chromosome I and NC_002506 for chromosome II), which is highly similar (>99.6% of VchM target sites are conserved). Reads that mapping to rRNA and tRNAs were excluded. A gene was included for differential expression analysis if it had more than one count per million reads (CPM = 1) in at least two samples. Differentially expressed genes (>2-fold differences, p-value <0.01, false discovery rates <0.2) were identified by the software program edgeR [70]. Expression differences for all genes in ∆*vchM* C6706 and O395 cells are located in S7 Table and S8 Table.

### Correlation analysis between motif counts and gene expression fold changes

For motif counts within gene coding regions, we first use linear regression (regress fold change on gene length) to get residuals after removing gene length effects (Fig 2B). Furthermore, to confirm that the correlation between RCCGGY count in gene body and gene expression fold change is not due to GC bias, or more generally less-specific motifs of RCCGGY, we use a linear regression to remove the effects of all less-specific motifs. For example, for RCCGGY, the less-specific motifs are RCCGG, CCGGY, RCCG, CCGG, CGGY, RCC, CCG, CGG, GGY, RC, CC, CG, GG, GY, R, C, G, Y. We observed partial correlation between motif count and gene expression fold change after removing effects of all less-specific motifs, as well as length of the gene. As shown in S4C Fig, only the RCCGGY motif has significant correlation with fold change.

The RCCGGY motif distribution within genes was determined using a custom Python script. Briefly, RCCGGY motifs in every gene were localized to windows corresponding to 5% of the coding length of the gene and the sites in each window enumerated.

### Transposon mutant library construction and sequencing

Transposon libraries were created in wildtype, *∆vchM*, *or ∆vchM∆rpoE V*. *cholerae*, and genomic DNA was purified and sequenced as previously described [23], with the exception that 10 ug of purified genomic DNA was sheared to ~350 bp fragments through acoustic disruption (Covaris, Woburn, MA, USA) for each DNA library. After sequencing and mapping, the read counts for every TA site were tallied and assigned to annotated genes or intergenic regions using custom scripts [23]. The raw read count data for all libraries can be found in S1 Table. Reads in the WT and mutant TIS libraries were normalized for differences in library saturation and read depth through simulation-based resampling and then subjected to Mann-Whitney U statistical tests as previously described in the ARTIST pipeline [66]. Candidates with significant p-values (<0.001) and > 5 fold differences in normalized read counts were considered as candidates for follow-up. In total, the WT, *∆vchM* and *∆vchM ∆rpoE* libraries contained ~650000 colonies with 118683, 103029, and 115845 unique transposon insertions detected from 3125378, 656980, 2709183 total mapped reads, representing insertions at 62%, 53% and 60% of all TA dinucleotides, respectively. The full ARTIST analyses for the *∆vchM* and *∆vchM ∆rpoE* experiments as well as the raw read counts data are found in S3, S4 and S5 Tables, respectively.

### Immunoblotting

Strains of interest were harvested at mid-exponential phase (OD 0.5), lysed directly in 1X NuPAGE LDS buffer (Novex) containing 6uM DTT, separated by NuPAGE Bis-Tris gel electrophoresis and transferred onto nitrocellulose using the iBlot system (Life Technologies). Blots were incubated with rabbit polyclonal antisera against σ^E^ or monoclonal antibody against RpoB (sc56766, Santa Cruz Biotechnology) in 5% milk in TBST. Horseradish-peroxidase conjugated secondary antibodies (Pierce) and Supersignal West Pico chemilumeniscent substrate (Pierce) were used to detect primary antibody signal. Blots were visualized on X-ray film, which was subsequently digitized on a FujiFilm FLA-5100 imager, and bands quantitated using MultiGuage V3.1 image analysis software.

### Quantitative RT-PCR

Overnight stationary cells were inoculated into 3 ml LB + Sm medium, grown at 37°C until mid-late exponential phase (OD 600 0.5–0.8), harvested and total RNA extracted with TRIzol reagent (Life Technologies). RNA was treated with Turbo DNase I for 30 min (Life Technologies) and subjected to qRT-PCR as previously described [71]. Briefly, 1 μg total RNA was used for the reverse transcription reaction with Superscript III first strand synthesis system with random hexamers (Life Technologies). The synthesized cDNA was subjected to real time-PCR amplification using the Fast SYBR Green Master Mix kit (Life Technologies) on the StepOnePlus platform (Life Technologies) using primers shown in S6 Table. The amplification data was analyzed by ΔΔCT method utilizing *rpoC* mRNA as internal control.

## Supporting Information

S1 Fig Presence of *vchM* in *V*. *cholerae* and non-cholera Vibrios. Blastn was used to evaluate whether *vca0198* exists in different *V*. *cholerae* and non-cholera strains. The nucleotide sequence of *vca0198* was mapped to all of 11 complete genomes of *V*. *cholerae* and 22 complete genomes of Vibrio non-cholerae in NCBI. The third column gives the percentage of *vca0198* that overlaps with different genomes. The hits with query cover less than 1% were omitted.
(PDF)

S2 Fig Enrichment and depletion of RCCGGY motifs in the *V*. *cholerae* genome. (A) The abundance of RCCGGY motifs across the genome (a concatenation of the two chromosomes) was plotted relative to chromosomal location to show the variability in regional prevalence. (B) The distance between RCCGGY motifs is shown for each adjacent pair of motifs along the concatenated chromosomes (arrayed along the X axis). The genomic location of *vchM* and of several σ^E-^regulating genes is shown. The lack of RCCGGY motifs within the regions surrounding σ^E-^regulating genes as well as *vchM* itself was highly significant (p-value ~4.8e-6). (C) The location of genes highlighted in S1B relative to neighboring RCCGGY motifs is shown in greater detail.
(PDF)

S3 Fig Bisulfite sequencing of the VchM 5mC methylome. (A) For >99.99% of the non-RCCGGY cytosine sites, the fraction of methylation estimated by Bisulfite sequencing was less than 20% (and most were<<20%). This contrasts with the findings regarding RCCGGY motifs, which are methylated on >20% of the DNA molecules at 99.8% of sites, and suggests that identification of 5mC sites by bisulfite sequencing had high specificity (>99.8%) and sensitivity (>99.8%). (B) The fractions of methylated RCCGGY motifs detected in stationary phase bacteria were plotted against the methylation frequencies of the same sites in exponentially growing bacteria.
(PDF)

S4 Fig RCCGGY motifs correlate with differential gene expression. (A) The presence of RCCGGY motifs within 200 bp of the transcriptional start sites (TSS) of all genes^28^ was correlated with their changes in gene expression in *∆vchM* cells. The correlation with a similar motif, RCATGY, serves as a negative control. The boxes represent the fold change of genes in the 25%-75% quartile with the median fold change shown as a line. The whiskers represent 1.5 fold of the interquartile range (the third quartile minus the first quartile) away from the box. (B) The fold changes in gene expression in *∆vchM* cells were compared before and after adjusting for GC content differences. (C) A linear regression was used to remove the effects of all less specific sub-motifs—RCCGG, CCGGY, RCCG, CCGG, CGGY, RCC, CCG, CGG, GGY, RC, CC, CG, GG, GY, R, C, G, Y—and the partial correlation between motif count and gene expression fold change was calculated. Only the RCCGGY motif had significant correlation with gene expression changes.(D) The relative location of intragenic RCCGGY motifs was enumerated for all genes (black bars) and for those found to be differentially expressed (red bars).
(PDF)

S5 Fig Insertion frequency within *deaD* in wt and *vchM V*. *cholerae*. The raw number of reads originating from insertions on the forward (red) or reverse strand (green) in wt and *∆vchM* insertion libraries are shown. All potential insertion sites (TA dinucleotides) are marked by black bars.
(PDF)

S6 Fig OmpU levels are not altered in *∆vchM* cells. The abundance of OmpU in wt, *∆vchM* and *∆ompU V*. *cholerae* was detected using western blotting. The abundance of OmpU in mutant strains (relative to in the wt strain) is shown above, and is based on normalization to the intensity of the non-specific band (n.s.).
(PDF)

S1 Table Differentially expressed genes between wt and ∆vchM C6706 cells. (XLSX)

S2 Table Genes that are differentially expressed between wt and ∆vchM mutants in C6706 and O395 backgrounds. (XLSX)

S3 Table Transposon-insertion sequencing experiment comparing ∆vchM and wildtype cells. (XLSX)

S4 Table Transposon-insertion sequencing experiment comparing ∆vchM ∆rpoE and ∆vchM cells. (XLSX)

S5 Table Raw Reads counts from transposon-insertion sequencing experiments. (XLSX)

S6 Table Primers used in this study. (XLSX)

S7 Table Gene expression differences between wt and ∆vchM C6706 cells. (XLSX)

S8 Table Gene expression differences between wt and ∆vchM O395 cells. (XLSX)
