# Correction: A Cytosine Methytransferase Modulates the Cell Envelope Stress Response in the Cholera Pathogen

**DOI:** 10.1371/journal.pgen.1005739

**Published:** 2015-12-11

**Authors:** Michael C. Chao, Shijia Zhu, Satoshi Kimura, Brigid M. Davis, Eric E. Schadt, Gang Fang, Matthew K. Waldor

The word ‘Methyltransferase’ is misspelled in the article title. The correct title is: A Cytosine Methyltransferase Modulates the Cell Envelope Stress Response in the Cholera Pathogen. The correct citation is: Chao MC, Zhu S, Kimura S, Davis BM, Schadt EE, Fang G, et al. (2015) A Cytosine Methyltransferase Modulates the Cell Envelope Stress Response in the Cholera Pathogen. PLoS Genet 11(11): e1005666. doi:10.1371/journal.pgen.1005666
