# Supplementary material for: A Cytosine Methytransferase Modulates the Cell Envelope Stress Response in the Cholera Pathogen
Source: PLoS Genet. 2015 Nov 20;11(11):e1005666. doi: 10.1371/journal.pgen.1005666 (PMC4654547; doi:10.1371/journal.pgen.1005666)
Supplement: S1 Fig — Blastn was used to evaluate whether vca0198 exists in different V. cholerae and non-cholera strains. The nucleotide sequence of vca0198 was mapped to all of 11 complete genomes of V. cholerae and 22 complete genomes of Vibrio non-cholerae in NCBI. The third column gives the percentage of vca0198 that overlaps with different genomes. The hits with query cover less than 1% were omitted. (PDF) [file pgen.1005666.s001.pdf]

Vibrio cholerae/ non-cholerae	Complete genome (Taxonomy ID)	Query cover
Vibrio Cholerae	Vibrio cholerae H1 (taxon:1093790)	100%
Vibrio Cholerae	Vibrio cholerae IEC224 (taxon:1134456)	100%
Vibrio Cholerae	Vibrio cholerae MS6 (taxon:1420885)	100%
Vibrio Cholerae	Vibrio cholerae O1 biovar El Tor str. N16961 (taxon:243277)	100%
Vibrio Cholerae	Vibrio cholerae O395 (taxon:345073)	100%
Vibrio Cholerae	Vibrio cholerae M66-2 (taxon:579112)	100%
Vibrio Cholerae	Vibrio cholerae MJ-1236 (taxon:593588)	100%
Vibrio Cholerae	Vibrio cholerae (taxon:666)	100%
Vibrio Cholerae	Vibrio cholerae O1 biovar El Tor (taxon:686)	100%
Vibrio Cholerae	Vibrio cholerae O1 str. 2010EL-1786 (taxon:914149)	100%
Vibrio Cholerae	Vibrio cholerae LMA3984-4 (taxon:935297)	2%
Vibrio non-Cholerae	Vibrio campbellii ATCC BAA-1116 (taxon:338187)	94%
Vibrio non-Cholerae	Vibrio vulnificus (taxon:672)	53%
Vibrio non-Cholerae	Vibrio sp. EJY3 (taxon:1116375)	2%
Vibrio non-Cholerae	Vibrio antiquarius (taxon:150340)	2%
Vibrio non-Cholerae	Vibrio vulnificus YJ016 (taxon:196600)	2%
Vibrio non-Cholerae	Vibrio vulnificus CMCP6 (taxon:216895)	2%
Vibrio non-Cholerae	Vibrio nigripulchritudo (taxon:28173)	2%
Vibrio non-Cholerae	Vibrio vulnificus MO6-24/O (taxon:914127)	2%
Vibrio non-Cholerae	Vibrio tubiashii ATCC 19109 (taxon:1051646)	1%
Vibrio non-Cholerae	Vibrio coralliilyticus (taxon:190893)	1%
Vibrio non-Cholerae	Vibrio parahaemolyticus RIMD 2210633 (taxon:223926)	1%
Vibrio non-Cholerae	Vibrio parahaemolyticus (taxon:670)	1%

Supplementary Figure S1
