# Supplementary figures and images for: A Cytosine Methytransferase Modulates the Cell Envelope Stress Response in the Cholera Pathogen
Source: PLoS Genet. 2015 Nov 20;11(11):e1005666. doi: 10.1371/journal.pgen.1005666 (PMC4654547; doi:10.1371/journal.pgen.1005666)

a

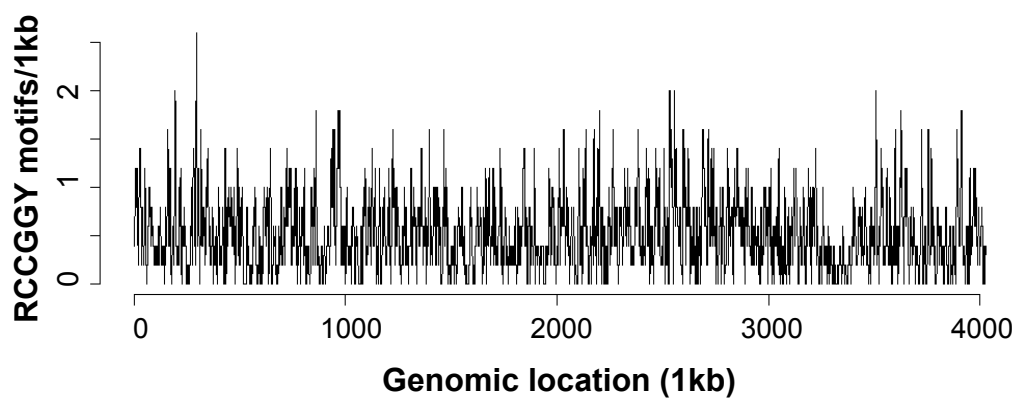

b

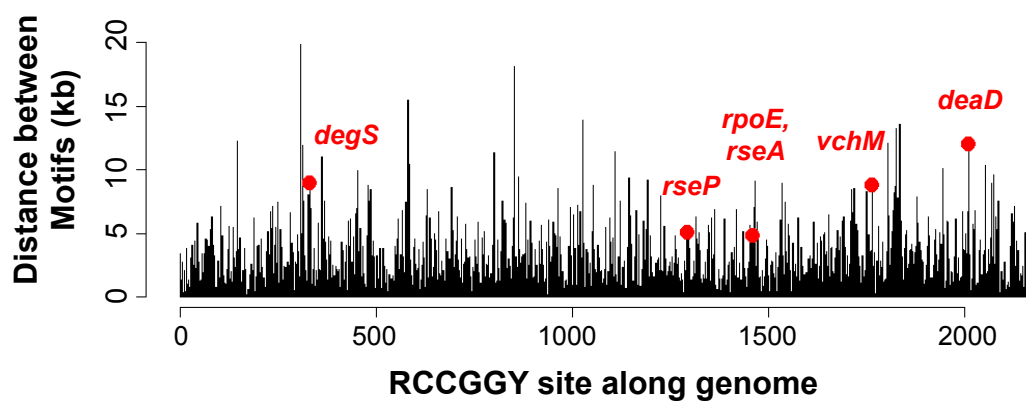

c

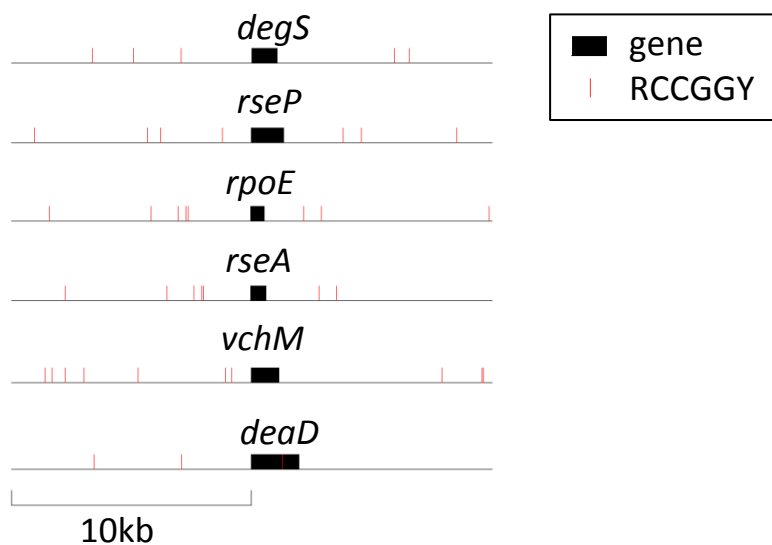

Supplementary Figure S2

Supplement: S2 Fig — (A) The abundance of RCCGGY motifs across the genome (a concatenation of the two chromosomes) was plotted relative to chromosomal location to show the variability in regional prevalence. (B) The distance between RCCGGY motifs is shown for each adjacent pair of motifs along the concatenated chromosomes (arrayed along the X axis). The genomic location of vchM and of several σE-regulating genes is shown. The lack of RCCGGY motifs within the regions surrounding σE-regulating genes as well as vchM itself was highly significant (p-value ~4.8e-6). (C) The location of genes highlighted in S1B relative to neighboring RCCGGY motifs is shown in greater detail. (PDF) [file pgen.1005666.s002.pdf]

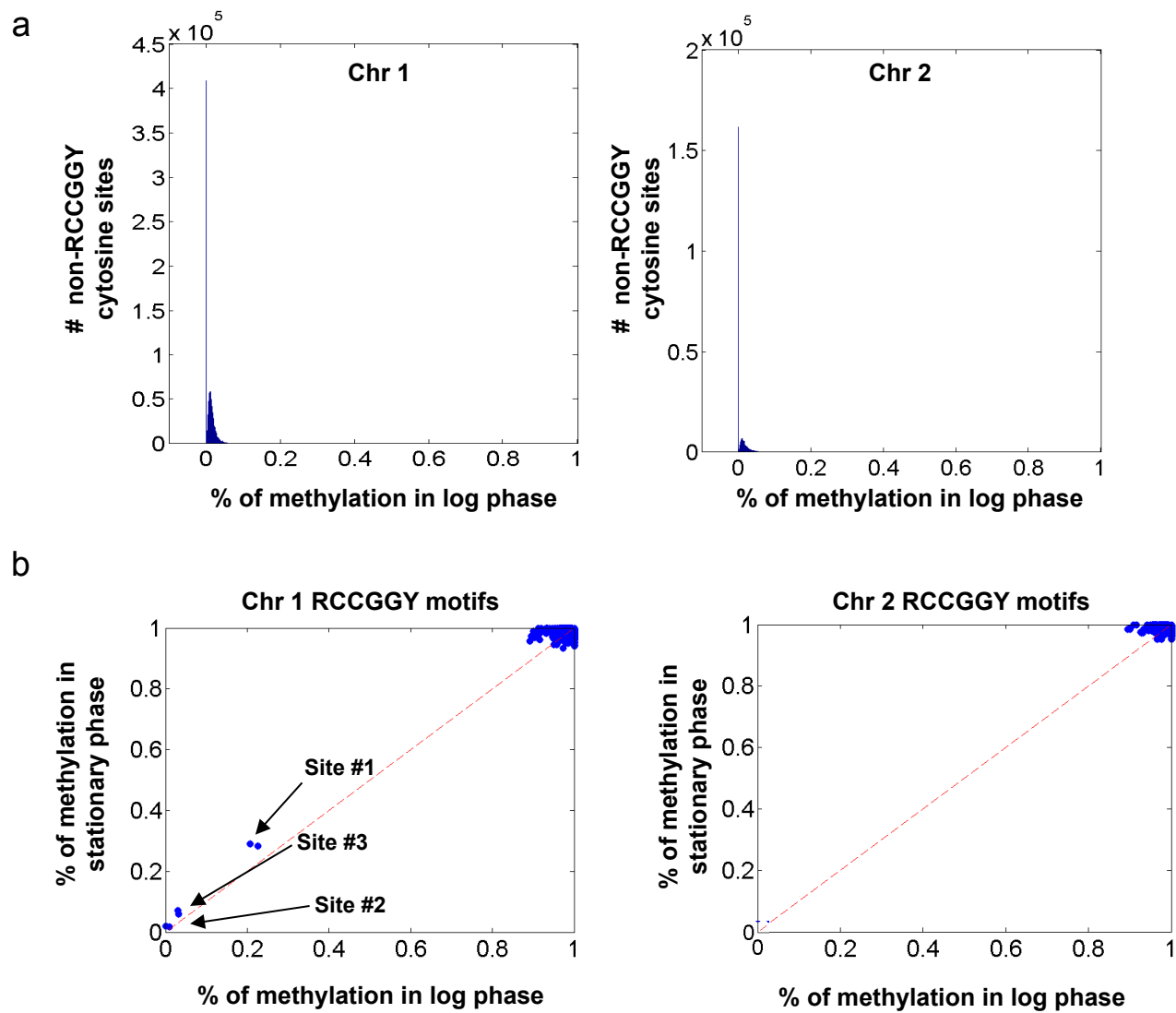

Supplementary Figure S3

Supplement: S3 Fig — (A) For >99.99% of the non-RCCGGY cytosine sites, the fraction of methylation estimated by Bisulfite sequencing was less than 20% (and most were<<20%). This contrasts with the findings regarding RCCGGY motifs, which are methylated on >20% of the DNA molecules at 99.8% of sites, and suggests that identification of 5mC sites by bisulfite sequencing had high specificity (>99.8%) and sensitivity (>99.8%). (B) The fractions of methylated RCCGGY motifs detected in stationary phase bacteria were plotted against the methylation frequencies of the same sites in exponentially growing bacteria. (PDF) [file pgen.1005666.s003.pdf]

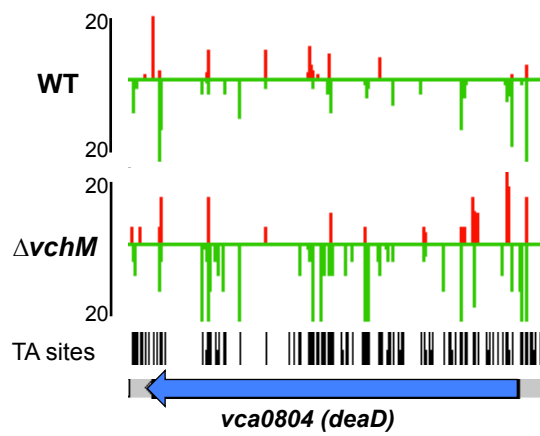

Supplementary Figure S5

Supplement: S5 Fig — The raw number of reads originating from insertions on the forward (red) or reverse strand (green) in wt and ∆vchM insertion libraries are shown. All potential insertion sites (TA dinucleotides) are marked by black bars. (PDF) [file pgen.1005666.s005.pdf]

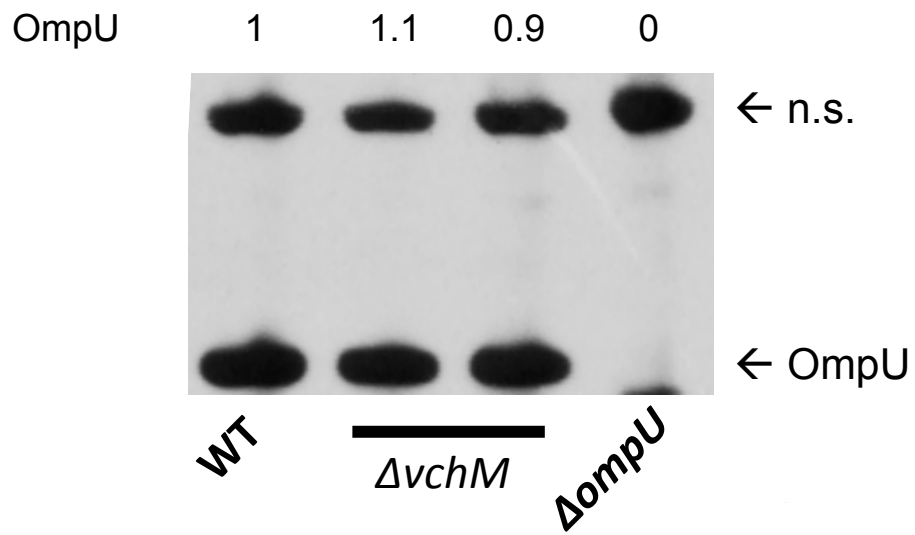

Supplementary Figure S6

Supplement: S6 Fig — The abundance of OmpU in wt, ∆vchM and ∆ompU V. cholerae was detected using western blotting. The abundance of OmpU in mutant strains (relative to in the wt strain) is shown above, and is based on normalization to the intensity of the non-specific band (n.s.). (PDF) [file pgen.1005666.s006.pdf]
