# Supplementary material for: A Cytosine Methytransferase Modulates the Cell Envelope Stress Response in the Cholera Pathogen
Source: PLoS Genet. 2015 Nov 20;11(11):e1005666. doi: 10.1371/journal.pgen.1005666 (PMC4654547; doi:10.1371/journal.pgen.1005666)
Supplement: S4 Fig — (A) The presence of RCCGGY motifs within 200 bp of the transcriptional start sites (TSS) of all genes28 was correlated with their changes in gene expression in ∆vchM cells. The correlation with a similar motif, RCATGY, serves as a negative control. The boxes represent the fold change of genes in the 25%-75% quartile with the median fold change shown as a line. The whiskers represent 1.5 fold of the interquartile range (the third quartile minus the first quartile) away from the box. (B) The fold changes in gene expression in ∆vchM cells were compared before and after adjusting for GC content differences. (C) A linear regression was used to remove the effects of all less specific sub-motifs—RCCGG, CCGGY, RCCG, CCGG, CGGY, RCC, CCG, CGG, GGY, RC, CC, CG, GG, GY, R, C, G, Y—and the partial correlation between motif count and gene expression fold change was calculated. Only the RCCGGY motif had significant correlation with gene expression changes.(D) The relative location of intragenic RCCGGY motifs was enumerated for all genes (black bars) and for those found to be differentially expressed (red bars). (PDF) [file pgen.1005666.s004.pdf]

a

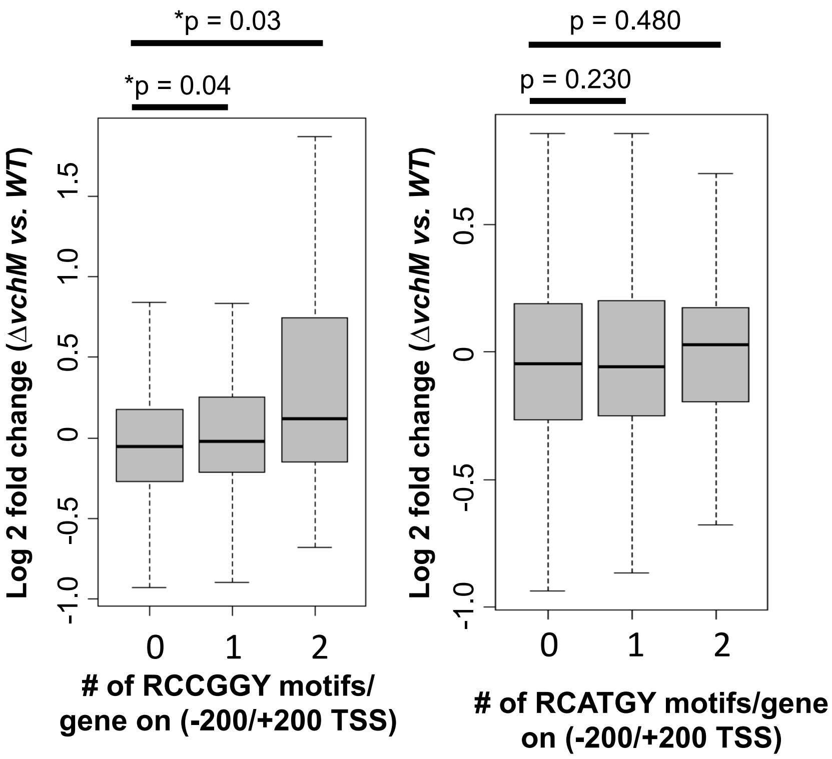

b

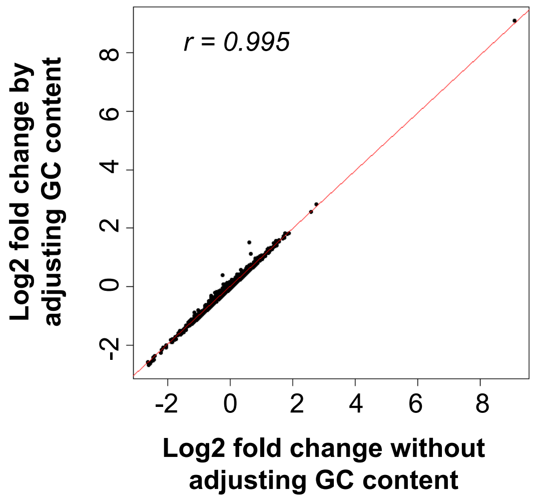

c

Motif	Partial correlation	P-value
RCCGGY	0.036	0.027
RCGCGY	0.016	0.34
RCCGGR	-0.0011	0.95
YCCGGR	-0.0038	0.81
GGCC	0.0096	0.55
GCGC	-0.012	0.46
RCATGY	0.0097	0.55

d

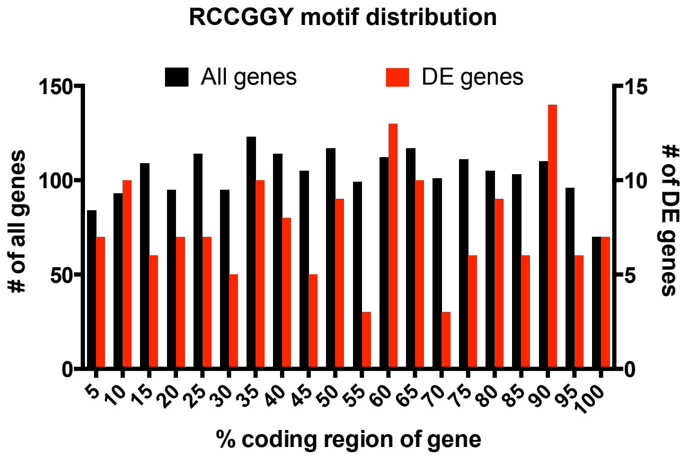

Supplementary Figure S4
